# The Incidence of Euphoria in Multiple Sclerosis: Artefact of Measure

**DOI:** 10.1155/2016/5738425

**Published:** 2016-06-13

**Authors:** Amy Duncan, Susan Malcolm-Smith, Ozayr Ameen, Mark Solms

**Affiliations:** Department of Psychology, University of Cape Town, Cape Town 8000, South Africa

## Abstract

*Background*. A subgroup of MS patients present with “euphoria.” Classical authors describe this symptom as the predominant mood state of these patients, while contemporary authors regard it as rare.* Objective*. This study aimed to address these discrepancies and investigate the contributions made by varying operational definitions and measurement instruments.* Methods*. One hundred MS patients and 100 matched controls completed the classical interview of Cottrell and Wilson and the modern Neuropsychiatric Inventory in a once-off interview.* Results*. The MS group demonstrated high frequencies of euphoria using the classical measure but low frequencies using the contemporary measure and definition. The matched control group demonstrated significantly higher rates than the MS group using the classical measure and lower rates than the MS group using the contemporary measure.* Conclusion*. The discrepancies in incidence rates of euphoria noted in the literature do not reflect a change in the incidence of euphoria in MS, but rather in the definition and operationalisation of “euphoria.” Furthermore, these results highlight the importance of characterising what represents pathological euphoria as well as the need for better definitions and instruments of measure.

## 1. Introduction

It has long been known that a subgroup of patients with MS show positive mood and optimism that is incongruous with their circumstances, as well as unawareness of increasing impairment. Reports of these symptoms, collectively referred to as “euphoria,” date back to the 1800s. A critical review of the historical development of these constructs has revealed a number of changes in the conceptual definitions since their first appearances in the literature, regarding the number of symptoms that constitute euphoria as well as the definitions of those types [1]. However, a change in incidence of these symptoms also appears to have occurred.

Charcot and Sigerson, circa 1877 [2], is well-known for his description of the cognitive and affective sequelae and the “stupid indifference” of MS patients, which he said referred to “most of the patients” (p. 194). In 1878, Wilk's [3, 4] characterisation of MS patients as happy and more likely to be found laughing than crying applied to “many patients,” and in the 1880s the accounts of Moxon and Gowers [3] of the positive mood and optimism of MS patients were “as a rule” and “especially frequent” (p. 244, 245). In 1904, Hoffman [5] stated that euphoria was a “characteristic feature of the mental state” of MS patients (p. 749).

These early reports gave the impression that pathological euphoria was the dominant mood state of patients with MS, and although they gave examples from particular cases, the claims made were often based on anecdotal observations, the total numbers of which were not readily reported. The 1920s saw the beginning of larger sample sizes being reported; however, the earlier suppositions concerning high rates of pathological euphoria continued to be confirmed. In 1922, Brown and Davis [6] found 71% (10/14) of their MS patients with “mental symptoms” to be euphoric (p. 629). In 1926, Cottrell and Wilson [7] found “euphoria sclerotica” (i.e., elevated positive mood) in 63%, “eutonia sclerotica” (i.e., a sense of physical well-being or an unawareness of physical deficit) in 84%, and “spes sclerotica” (i.e., optimism for the future) in 84% of their 100 MS patients. Even as late as 1943 Sugar and Nadell [8], who attempted to replicate the earlier findings of Cottrell and Wilson, found euphoria sclerotica in 53.6%, eutonia sclerotica in 50%, and spes sclerotica in 50% of their 28 MS patients.

A shift in research focus appeared to occur in the latter half of the 20th century, and when interest in euphoria in MS resumed at the turn of the 21st century, a change appeared to have occurred. Today the symptom is considered to be dramatically less common than in the classical literature. With specific reference to the main contemporary studies investigating the frequency of euphoria in MS, Figved et al. [9] demonstrated pathological euphoria in only 4.7% (*N* = 86) and Diaz-Olavarrieta et al. [10] in 13% (*N* = 44) of their samples. Fishman et al. [11] demonstrated pathological euphoria in 14.6% of their sample (*N* = 75); however, they considered their euphoria/disinhibition factor, which they demonstrated in only 9% of their sample, to be more representative of the classic euphoria sclerotica.

It therefore becomes apparent that discrepancies exist regarding the incidence rates of pathological euphoria reported throughout the history of this phenomenon. Although reasons, such as differences in disease duration across samples [12, 13], inadequate screening among early reports to rule out other diseases such as neurosyphilis [3, 14], and differences in definitions and measurement instruments [3, 12–16], have been postulated, no study has yet specifically addressed the issue of differing incidence rates or empirically investigated factors which may directly impact this question.

The current study aimed to address this perplexing situation. We hypothesised that the change in incidence could be related to the demonstrated change in conceptual definition and also to a change in the operational definitions of the symptoms (i.e., the instruments used to measure these symptoms) rather than a change in the MS population itself and that high rates of pathological euphoria could be replicated by using a classical measure and low rates could be replicated by using a contemporary measure. Such an investigation is important in better understanding “euphoria” as well as the frequencies with which it can be found in MS patients.

## 2. Materials and Methods

The study obtained ethical approval from the Research Ethics Committee of the Faculty of Health Sciences, University of Cape Town. One hundred patients with a diagnosis of MS and an informant known to each patient as well as 100 matched healthy controls (HC) and an informant known to each control were recruited for voluntary participation. The characteristics of the participants are presented in Table 1.

### 2.1. Classical Measure

The original interview schedule published by Cottrell and Wilson [7] (see Appendix) was included as a representation of the classical operational definition and measurement instrument. It can be found in the public domain and assesses three types of euphoria: euphoria sclerotica (positive mood), eutonia sclerotica (physical well-being and unawareness of physical deficit), and spes sclerotica (optimism). The questions, which appear to elicit subtle, mild symptoms, include, for example, “Do you feel consistently cheerful or happy?”, “Are you conscious of any pleasant or unpleasant sensation in your body as a whole or a part?”, “Is the feeling one of bodily ease?”, and “Are you naturally optimistic?”. Yes/no answers are given by the participant.

In their article, Cottrell and Wilson listed their questions; however, they did not specify the rating criteria imposed to determine the frequencies found for each of the three types. Rating criteria, used by 3 independent raters, were therefore created for this study. Initially, a present/absent criterion was used, but after confusion was voiced by the raters regarding a mixed picture in some answers, each of the 3 raters were rather asked to score each type of euphoria a “2” if all answers pertaining to the specific type of euphoria were affirmative and the raters therefore considered the symptom to be definitely present; a “1” if the answers were mixed with only some answers being affirmative and the raters were of the opinion that the symptom was possibly present; and a “0” if no answers were affirmative and the raters therefore considered the symptom to be absent. The average of the three raters was then calculated and rounded up or down to the nearest whole number (“2,” “1,” or “0”), as per the rating criteria. Interrater reliability was calculated for each of the three types: (a) ICC = .82 (euphoria sclerotica), (b) ICC = .60 (eutonia sclerotica), and (c) ICC = .90 (spes sclerotica).

### 2.2. Contemporary Measure

The question referring to euphoria in the Neuropsychiatric Inventory (NPI) [17] was included as a representation of the measure (i.e., operational definition) most often used in contemporary euphoria research [9–11]. In contrast to the classical measure, the NPI only refers to positive mood (i.e., one instead of three types of euphoria) and does not address aspects relating to physical well-being/unawareness of physical deficit or optimism. Further, it appears to focus more on extreme symptoms: “does the patient seem too cheerful or too happy for no reason? I do not mean the normal happiness that comes from seeing friends, receiving presents, or spending time with family members. I am asking if the patient has a persistent and* abnormally* good mood or finds humour where others do not” [17].

Standard administration requires an informant known to the patient to answer the euphoria question by providing a yes/no response about the patient. However, in this study, participants were also asked to self-report euphoria by answering the same question about themselves.

## 3. Results and Discussion

### 3.1. Results

The frequencies of the three types of euphoria, according to the classical measure, within the two groups, as well as a comparison between MS and HC groups for the “total” presence of these variables are depicted in Tables 2 and 3.

In terms of the contemporary measure, 11% of informants reported their MS loved one as being pathologically euphoric, and 16% of participants self-reported the experience of pathological euphoria. With regard to the healthy control group, 4% of informants reported their healthy loved one as being pathologically euphoric, and 4% of healthy controls self-reported the experience of pathological euphoria. Comparisons between MS and HC groups are presented in Table 4.

A summary of the current results, along with previously reported frequencies using the same measures, are presented in Table 5. As other studies using the NPI only use the standard administration method, only informant reported euphoria is included.

### 3.2. Discussion

The aim of this study was to address the discrepancies noted in the literature regarding incidence rates of pathological euphoria in MS patients. It was the first study of its kind to specifically investigate these discrepancies and the impact of different measures of euphoria, by using different operationalisations on a single sample of MS patients.

Since no guidance was given regarding the interpretation of the classical measure, if one presumes that the current study's criteria employed for a definite presence of the symptom were too strict and one includes the possibly present cases, where some of the answers were affirmative a the particular type of euphoria, euphoria sclerotica was found in 63%, eutonia sclerotica in 48%, and spes sclerotica in 70% of the current sample of 100 MS patients. This is comparable to the 63% euphoria sclerotica, 84% eutonia sclerotica, and 84% spes sclerotica found by Cottrell and Wilson [7] and with the 53.6% euphoria sclerotica, 50% eutonia sclerotica, and 50% spes sclerotica reported by Sugar and Nadell [8], who later attempted to replicate the original study. Thus, high frequencies of the euphoria types, albeit with slight interpretation, were found by the current study when using the more subtle classical operational definition and measure. Even when one only considers the definite cases, the frequencies remain higher than those which are considered the norm today.

By contrast, low rates of pathological euphoria (in terms of only abnormal positive mood) were demonstrated by this study when using the NPI; that is, only 11% of informants rated their MS loved ones as euphoric according to the NPI definition. This is similar to the 13% found by Diaz-Olavarrieta et al. [10] and the 14.6% found by Fishman et al. [11]. Thus, low incidences of pathological euphoria were found when using the more severe contemporary measure (and operational definition).

As the same MS patients (and their informants) were tested on both measures at the same time and a dramatic change in the patients' mood is unlikely to have occurred to account for the discrepancy seen, the current results appear to demonstrate that pathological euphoric symptoms can be found in relatively high frequencies when described in more mild, subtle terms, but in relatively low frequencies when defined more severely.

Furthermore, it is important to note that the classical measure was based on a self-report questionnaire/interview, while the NPI was informant-based. However, even when patients were asked to rate themselves according to the NPI euphoria question, only 16% (a similarly low rate) self-reported pathological euphoria according to the contemporary definition, negating the possible confounding influence of self- versus informant-reporting on the inconsistency between classical versus contemporary incidence rates.

In our previous work, we established that a change in the conceptual definition of pathological euphoria appears to have occurred over the last 100 years [1]. In this paper, we broaden that notion to include that a change in operational definition appears to have also taken place. The classical instrument used in this study measures pathological euphoric mood, for example, by asking, “Do you feel consistently cheerful or happy?” [7], while the modern NPI refers to pathological euphoric mood by asking if the patient has “a persistent and abnormally good mood” [17]. These clearly represent different mood states and the findings of the current study therefore imply that different measuring instruments (based on operational definitions which also appear to have changed) have influenced the rates of pathological euphoria reported throughout the MS literature and that discrepancies between high classical and low contemporary rates could be the result of measurements artefacts [18, 19], rather than any change in reality. Thus, not only is pathological euphoria described differently today but the new definitions have also influenced the rates at which it is found.

What caused the change in definition and incidence is beyond the scope of this paper. However, one possible contributing factor could have been experimenter bias, as classical authors appeared to be biased towards detecting the presence of pathological euphoria, believing it to be the “dominant mood state” of MS patients, while contemporary authors appear to be biased against it, believing it to be rare. As their operational definition is considerably more subtle and their rating criteria were not objectively stated, the early authors Cottrell and Wilson may represent a prime example of this bias by being too inclusive and classifying patients whose answers were mixed as euphoric. However, it is important to note that although Benedict et al. and Fishman et al. justify their use of the NPI in euphoria research based on the fact that it is a standardised measurement instrument [11, 20], no literature exists criticising the more subtle classical operational definition in favour of a more severe one, thereby supporting a need for the change.

Other possibilities, relating to bias, also exist which may have influenced incidence rates. MS was not a treatable condition during the period in which higher rates of pathological euphoria were found. A selection bias may have occurred with the researchers and neurologists investigating pathological euphoria in that perhaps those MS patients coming to their attention were more hopeful about their prognosis and more likely to seek out care than those MS patients who had accepted their fate. Conversely, it may not have been those MS patients who sought out treatment but rather those who required treatment who were brought to the attention of the early researchers. We now know that pathological euphoria in MS has been found to correlate with dementia [11, 13]. It may be possible that the MS patients who came into contact with the neurologists and other researchers in early times did so because they required psychiatric attention due to dementia. This seems unlikely in relation to the study by Cottrell and Wilson, due to the relatively short disease duration among their sample (i.e., 51% of their sample had had MS for 5 years or less) [7] and the findings that both euphoria and dementia correlate with advanced disease [9, 13]. However, the possibility of MS patients coming to the attention of neurologists due to their needing psychiatric care may be a possible factor leading to higher rates of pathological euphoria in other early MS samples.

Regardless of what caused the change, these findings leave open the question as to which rates and associated definitions are correct. The answer, however, may not be a clear cut choice between the two. Since pathological euphoria in MS has been demonstrated to occur in patients with cerebral and not spinal cord involvement, it is typically regarded as an organic symptom and not a psychological reaction to the disease [21]. Furthermore, pathological euphoria (in terms of the NPI definition) has been found to correlate with both lesion load and atrophy [22], grey matter atrophy [23], and frontotemporal changes on MRI [10]; and some have proposed it may be due either to a disconnection of the frontal cortex and limbic structures by white matter lesions [11] or to grey matter atrophy of the prefrontal cortex [11, 23]. Thus, the way in which euphoria is defined and measured and the question of what represents a pathological symptom are of vital importance when considering which definition best represents this symptom. With regard to the classical measure, how can we be sure that the subtle questions included and the “symptom” measured truly represent a pathological symptom? Equally, when considering the more severe modern operational definition, can we be sure we are not excluding some patients experiencing slightly milder yet still pathological euphoria?

In order to address this issue, the frequency of euphoria was compared among MS patients and a matched sample of healthy controls in this study. Unlike other modern studies, a control group equal in size to the patient group was used and 4% of HCs were reported as having or self-reported the experience of (contemporary NPI) euphoria. This is in contrast to the 0% found by Diaz-Olavarrieta et al. and Fishman et al. who used more limited control samples of 25 each in comparison to their patient samples that were at least double that [10, 11]. In this study, significant differences between MS patients and HCs were demonstrated only for self-reported euphoria (in terms of the modern NPI) with higher frequencies of MS patients experiencing euphoria (*p* = .005). Although higher frequencies of MS patients than HC were identified as euphoric using the traditional informant-based administration, this comparison did not yield significant results (*p* = .060). Since pathology can be defined implicitly as a significant difference between patients and controls, one therefore cannot assume that the NPI is effectively identifying a pathological symptom, although the results imply that the measure is measuring something close to pathological as more MS patients than HCs identified themselves and were identified by their loved ones as being euphoric.

The earlier studies of Cottrell and Wilson and Sugar and Nadell, by contrast, did not include a control group of any kind. However, the current study identified significantly fewer MS patients than matched HCs as having euphoria as defined and measured by the classical measure (all *p* values < .0001). One might take this to mean that the classical operational definition (and measure) is even less likely than the NPI to represent a pathological symptom. Although both measures use a present/absent approach, it may be that the method of analysis for the classical measure is too inclusive and that those that were identified as “definitely euphoric” represent a more pathological representation of the symptom. However, the discrepancies between MS and HC participants for definite euphoria were also significant, with fewer MS patients demonstrating definite euphoria than HC (euphoria sclerotica: *p* = .008; eutonia, and spes sclerotica: *p* < .0001). Thus, when compared to the contemporary measure, the classical measure certainly appears to be ill-representative of pathological euphoria.

This may mean that the interview of Cottrell and Wilson is not valid and that it simply does not elicit and measure pathological euphoria. Issues around the interpretability of this measure and the lack of direction in the original paper regarding rating criteria have already been raised. Further, the subtle nature of the questions, such as “Do you feel consistently cheerful or happy?”, has also been noted. While questions that are slightly more subtle than the “persistent and abnormally good mood” of the NPI may be appropriate, the questions of Cottrell and Wilson may simply be too general, reflective of basic personality traits rather than anything pathological. Indeed, in research with MS patients using the Five Factor Model and the revised version of the NEO Personality Inventory (which both measure the same five factors of personality: neuroticism, extraversion, openness to experience, agreeableness, and conscientiousness), a subset of MS patients have been found to demonstrate elevated neuroticism, as well as reduced agreeableness and conscientiousness [20]. Cottrell and Wilson's question, “Are you naturally optimistic?”, is not reflective of psychopathology, but the item would likely load on the neuroticism factor in a personality trait study. Thus, the potential invalidity of Cottrell and Wilson's interview as a measure of pathological euphoria appears to be a more likely reason for the higher rates of euphoria among HCs than MS patients.

However, of interest is that the elevated neuroticism and reduced agreeableness and conscientiousness within this subset of MS patients have been found to correlate with a euphoria/disinhibition factor identified using the NPI [11]. Thus, while the Cottrell and Wilson interview may not measure pathological euphoria, it may nevertheless measure personality traits consistent with euphoric MS patients.

Although the NPI may be more appropriate than mild, subtle definitions, significant differences were not identified between MS and HC groups for informant reported euphoria, the standard way in which this test is administered. This may imply that the modern NPI definition is not quite as severe (or exclusive) as we, the current authors, might have believed and that even more severe definitions are required to determine a cut-off rate that is deemed pathological (when comparing patients and controls). Although it may have been favoured due to its being standardised, other measures may exist that better encompass euphoria, and the popularity of this measure may simply be due to a number of papers being published using it to represent euphoria, which has, in turn, led to the perpetuation of perhaps erroneous incidence rates of euphoria.

There may still, however, be use for more subtle definitions, somewhere between mild and harshly defined euphoria. Since modern research has identified that NPI euphoria tends to occur later in the disease along with significant disability [9, 11, 13], the identification of more subtle forms may allow for the prediction of which patients will develop more severe forms as the disease progresses and the representation of pathological euphoria in MS on a continuum from more subtle to severe forms may be of clinical significance. Further, although abnormality may be defined as a significant difference between patients and controls, pathology can also be defined in terms of the extent to which a symptom impacts the patient's ability to function, and even subtle forms of euphoria may impact the care and treatment of patients and affect their families.

In our review of the change in conceptual definition of euphoria [1], we noted the need for clear, reliable, and widely recognised conceptual definitions of euphoria and we extend this here to consistent and accepted means of measuring these complex constructs as well as a comprehensive understanding of what characterises a pathological representation of this symptom. Without standardised measurement instruments we will continue to face unreliable incidence rates that cannot be compared and will continue to either under- or overreport the incidence of this interesting cluster of neuropsychiatric symptoms.

## 4. Conclusions

The discrepancies in incidence rates of pathological euphoria noted between the historic and contemporary literatures do not reflect a change in the incidence of euphoria in MS, but rather in the definition and operationalisation of the term. Furthermore, measures often used in euphoric literature may be ill-representative of a symptom that could be considered pathological.

## Figures and Tables

**Table 1:** Sociodemographic and medical characteristics of the MS patients and healthy controls (*N* = 100).

Characteristic	MS	HC	*t* (df = 198)/*χ* ^2^ (df = 1)	*p* (2-tailed/2-sided)	95% CI	
					LL	UL
Gender, male : female	14 : 86	14 : 86	.0001	1.000		
Race, Caucasian : Coloured/Indian	71 : 29	73 : 27	.099	.753		
Age	44.49 ± 11.17 (19–72)	43.75 ± 11.02 (19–69)	−.472	.638	−3.83	2.35
Education	13.18 ± 1.65 (8–15)	13.40 ± 1.50 (8–15)	.984	.326	−0.22	0.66
Income	R26,006.51 ± R22,536.54 (R1,200.50–R153,601.00)	R26,993.51 ± R21,480.21 (R1,200.50–R153,601.00)	.317	.752	−5,152.58	7,126.58
Disease duration	9.57 ± 7.5 (0–42)					
Disease course, RRMS : PPMS : SPMS	75 : 10 : 15					

*Note*. All continuous data are represented as means and standard deviations, with ranges in parentheses. In defining “race,” apartheid era classifications continue to be used in South Africa to designate previously disadvantaged groups. “Coloured” refers to individuals with a mixed race background. “Education” represents highest level of education in years. “Income” represents monthly household income. “Disease duration” was measured from year of diagnosis, represented in years.

RRMS = Relapsing-Remitting Multiple Sclerosis; PPMS: Primary Progressive Multiple Sclerosis; SPMS = Secondary Progressive Multiple Sclerosis.

**Table 2:** Frequencies of euphoria sclerotica, eutonia sclerotica, and spes sclerotica, according to the classical interview of Cottrell and Wilson, among the MS patients and healthy controls (*N* = 200).

Frequency	Euphoria sclerotica (positive mood)						Eutonia sclerotica (physical well-being/unawareness of physical deficit)						Spes sclerotica (optimism)					
	MS			HC			MS			HC			MS			HC		
	Def.	Pos.	Tot.	Def.	Pos.	Tot.	Def.	Pos.	Tot.	Def.	Pos.	Tot.	Def.	Pos.	Tot.	Def.	Pos.	Tot.
Ave. ratings	21%	42%	**63%**	38%	48%	**86%**	6%	42%	**48%**	36%	54%	**90%**	37%	33%	**70%**	70%	24%	**94%**

*Note*. Total frequencies are presented in bold font. Def. = definitely present (all answers affirmative); Pos. = possibly present (some answers affirmative); Tot. = total (definitely present plus possibly present frequencies).

**Table 3:** Comparison between MS patients and healthy controls of number of participants with classical euphoria (*N* = 200).

Euphoric symptom	MS	HC	*χ* ^2^ (df = 1)	*p* (2-sided)
Euphoria sclerotica				
Total, present : absent	63 : 37	86 : 14	13.923	**<.0001**
Definite, present : absent	21 : 79	38 : 62	6.948	**.008**
Eutonia sclerotica				
Total, present : absent	48 : 52	90 : 10	41.234	**<.0001**
Definite, present : absent	6 : 94	36 : 64	27.125	**<.0001**
Spes sclerotica				
Total, present : absent	70 : 30	94 : 6	19.512	**<.0001**
Definite, present : absent	37 : 63	70 : 30	21.887	**<.0001**

*Note*. Significant results are presented in bold font.

**Table 4:** Comparison between MS patients and healthy controls of number of participants with contemporary euphoria (*N* = 200).

Euphoric symptom	MS	HC	*χ* ^2^ (df = 1)	*p* (2-sided)
Euphoria (informant-reported), present : absent	11 : 89	4 : 96	3.532	.060
Euphoria (self-reported), present : absent	16 : 84	4 : 96	8.000	**.005**

*Note*. Significant results are presented in bold font.

**Table 5:** Previous and current frequencies of euphoria sclerotica, eutonia sclerotica, and spes sclerotica among MS participants and healthy controls.

Frequency	MS			HC		
	Euphoria sclerotica (positive mood)	Eutonia sclerotica (physical well-being/unawareness of physical deficit)	Spes sclerotica (optimism)	Euphoria sclerotica (positive mood)	Eutonia sclerotica (physical well-being/unawareness of physical deficit)	Spes sclerotica (optimism)
Classical measure						
Previous findings	63% [7] (*N* = 100)	84% [7] (*N* = 100)	84% [7] (*N* = 100)	—	—	—
	53.6% [8] (*N* = 28)	50% [8] (*N* = 28)	50% [8] (*N* = 28)	—	—	—
*Current findings*	63% (**N** = 100)	48% (**N** = 100)	70% (**N** = 100)	86% (**N** = 100)	90% (**N** = 100)	94% (**N** = 100)

Contemporary measure						
Previous findings	4.7% [9] (*N* = 86)	—	—	—	—	—
	13% [10] (*N* = 44)	—	—	0% (*N* = 25)	—	—
	14.6% [11] (*N* = 75)			0% (*N* = 25)		
*Current findings*	11% (**N** = 100)	**—**	**—**	4% (**N** = 100)	**—**	**—**

*Note.* The findings of this study are presented in bold font.

## Appendix

### Interview Schedule of Cottrell and Wilson (1926)

*I. Emotional Content*

Describe in a few words your general or usual mood:
Do you feel consistently cheerful or happy?
Do you feel consistently sad or unhappy?
Are you naturally optimistic?
Are you naturally pessimistic?
Are you aware of any alteration in either respect since the onset of the illness?
Are you optimistic or pessimistic in reference to your disease?
Do you change readily from a feeling or cheerfulness to one of sadness, and vice versa?
Are you easily amused –
  By what you see?
  By what you hear?
  By what you read?
Are you easily depressed –
  By what you see?
  By what you hear?
  By what you read?
Are you moods fleeting or apt to last for some time?
Any change in this respect from formerly?
Are you naturally phlegmatic or indifferent?
Are you anxious or worried?
Are you irritable?
Do you easily lose your temper?
Are you different in mood in any of these respects from what you were one, two, five, ten, twenty years ago, or before the commencement of the illness?

*II. Psychical Determinants*

Are your thoughts consistently pleasant?
Are your thoughts amusing?
Are you inclined to daydream, to live in the future, to live in an ideal world, or to live in the past?
Are your thoughts consistently unpleasant, serious, sombre?
Are you inclined to ruminate on unpleasant subjects?
Are your thoughts depressing?
Are you inclined to worry about yourself?
Do you dream?
Are the dreams pleasant or unpleasant?

*III. Physical Determinants*

Describe your bodily feeling as a whole.
Are you conscious of any pleasant or unpleasant sensation in your body as a whole or a part?
Do you feel tired or fatigued?
Do you feel relaxed?
Do you feel sleepy?
Is the feeling one of bodily ease? Is the feeling one of contentment?
Is the feeling one of pleasure?
Is your general feeling one of malaise?
Do you feel tense?
Do you feel nervous or jumpy?
Have you any feeling or pain, aching, soreness?
Are you restless?
Does the performance of normal bodily functions produce pleasant or unpleasant sensations?

*IV. Affective Conduct*

Do you laugh easily?
Do you laugh without adequate cause?
Do you cry easily?
Do you cry without adequate cause?
Is your outward expression a reliable gauge of your inward feeling?
Can you control the expression of your feeling?
Are you different in any of these respects from what you were one, two, five, ten, twenty years ago, or before the commencement of the illness?
